# Epidemiological Trends of Malaria in Five Years and under Children of Nsanje District in Malawi, 2015–2019

**DOI:** 10.3390/ijerph182312784

**Published:** 2021-12-03

**Authors:** Theodore Gondwe, Yongi Yang, Simeon Yosefe, Maisa Kasanga, Griffin Mulula, Mphatso Prince Luwemba, Annie Jere, Victor Daka, Tobela Mudenda

**Affiliations:** 1 Department of Epidemiology and Biostatistics, College of Public Health, Zhengzhou University, Zhengzhou 450001, China; theodoregondwe@gmail.com (T.G.); kasangaanita@gmail.com (M.K.); 2 Department of Planning and Policy Development, Ministry of Health Malawi, Lilongwe 30377, Malawi; cyrilyosefe@gmail.com; 3 Department of Education Planning, Ministry of Education Malawi, Lilongwe 328, Malawi; gmulula@gmail.com; 4 Mathematical Department, Chancellor College, University of Malawi, Zomba 280, Malawi; luwembamphatso@gmail.com; 5 School of Engineering, Malawi University of Business and Applied Sciences, Blantyre 303, Malawi; ajere@mubas.ac.mw; 6 Public Health Department, Michael Chilufya Sata School of Medicine, Copperbelt University, Ndola 21692, Zambia; dakavictorm@gmail.com; 7 Pathology Department, Ndola Teaching Hospital, Ndola 10101, Zambia; mudendatobela@gmail.com

**Keywords:** malaria incidence, time series, SARIMA

## Abstract

Background: Malaria continues to be a major public health problem in Malawi and the greatest load of mortality and morbidity occurs in children five years and under. However, there is no information yet regarding trends and predictions of malaria incidence in children five years and under at district hospital level, particularly at Nsanje district hospital. Aim: Therefore, this study aimed at investigating the trends of malaria morbidity and mortality in order to design appropriate interventions on the best approach to contain the disease in the near future. Methodology: Trend analysis of malaria morbidity and mortality together with time series analysis using the SARIMA (Seasonal Autoregressive Integrated Moving Average) model was used to predict malaria incidence in Nsanje district. Results: The SARIMA model used malaria cases from 2015 to 2019 and created the best model to forecast the malaria cases in Nsanje from 2020 to 2022. An SARIMA (0, 1, 2) (0,1,1)_12_ was suitable for forecasting the incidence of malaria for Nsanje. Conclusion: The mortality and morbidity trend showed that malaria cases were growing at a fluctuating rate at Nsanje district hospital. The relative errors between the actual values and predicted values indicated that the predicted values matched the actual values well. Therefore, the model proved that it was adequate to forecast monthly malaria cases and it had a good fit, hence, was appropriate for this study

## 1. Introduction

There are 106 countries in the world at risk of the transmission of malaria infection where 216 million estimated malaria cases occurred in 2010, 81% of which were reported in the African Region, followed by South East Asia (13%) and the Eastern Mediterranean Region (5%) [1]. The total number of malaria deaths was estimated to be 655,000 in 2010, 91% of whom occurred in the African Region, 6% in South East Asia and 3% in the Eastern Mediterranean Region [2]. Malaria is a severe disease that is caused by parasites of the genus plasmodium, which is transmitted to humans by a bite of an infected female mosquito of the species anopheles. Plasmodium are global pathogens with a complex life cycle alternating between female Anopheles mosquitoes and vertebrate hosts that require the formation of unique zoite forms to invade different cell types at specific stages [3]. Malaria in human beings is mainly caused by six types of plasmodium species namely, *Plasmodium falciparum*, *Plasmodium vivax*, *Plasmodium malariae*, *Plasmodium ovale Curtis, plasmodium knowlesi* and *P. ovale wallikeri* [4]. Most of the malaria episodes are caused by *Plasmodium falciparum* (*P. falciparum*), which is the agent of the most severe and fatal malaria disease [2]. The African Region accounts for an estimated 92% of all malaria deaths from *P. falciparum* and *Plasmodium vivax* (*P. vivax*) [5].

As in most of sub-Saharan Africa (SSA), children under the age of five years bear the highest burden of malaria [6]. Every year the disease results in 300 million to 500 million clinical cases and causes more than one million deaths in children. The latter accounts for a dying rate of nearly 3000 deaths per day in children under five [7]. Furthermore, in Africa, some children suffer an acute attack of cerebral malaria that quickly leads to coma and death; others succumb to severe anaemia that follows repeated infections [8].

In order to control the morbidity and mortality of malaria, the governments of Zambia and Ghana have strengthened their health services for young children and collaborated with other key programmes for child survival, such as the Expanded Programme on Immunization, nutrition and the Integrated Management of Childhood Illness. The latter programs have led to the effective prevention and control of malaria in the two countries [9]. However, in countries such as Malawi, the burden of malaria still prevails. Every Malawian resident lives in a region of high malaria transmission, defined as greater than one case per 1000 residents [10]. According to [11], malaria is endemic in Malawi and transmission is higher in areas with high temperatures, low altitude and lower shire areas. Chikwawa is located in the lower shire valley where *p. falciparum* and anopheles thrive with an estimated entomological inoculation rate (EIR) for *p. falciparum* of 172 infective bites. Since Chikwawa is a neighboring district to Nsanje and it is located in the lower shire valley, Nsanje also holds the same environment for mosquito breeding. Supporting the latter fact, [12] states that in close to half (46%) of the districts in Malawi, greater than 75% of the population resides in areas with >40% predicted prevalence, with the highest prevalence found in Nsanje (42%). As a result. Nsanje is one of the areas with high transmission intensity of malaria in Malawi [12].

The incidence of Malaria displays a cyclic pattern, time series models are the most widely used models for forecasting diseases that show a cyclic pattern [5]. Time series analysis has the advantage of predicting incidence and it is characterized by the number of patients in the past and responds by predicting the number of patients in the future [13]. Malaria has high transmissibility and seasonality, and this makes SARIMA models the best fit for the data because of the high predictive power the model has [4]. There are several studies on the trend analysis of malaria in Africa: [14] used SARIMA (0, 1, 1) (0, 1, 1)_12_ to predict monthly malaria cases in KwaZulu-Natal in South Africa, while [15] used SARIMA (1, 0, 0) (0, 0, 0)_12_ to predict the effects of rainfall on malaria incidence in Eritrea.

Although infants have been considered to be relatively protected against malaria, there has been a substantial burden of the disease in Malawian infants during their first five years, with 10% of children under five years of age having been hospitalized with malaria [16]. A study conducted by [17], states that the sustainability of plasmodium parasites is only possible when the parasites infect red blood cells of their human host According to [18], in 2015, malaria incidence was at 380 cases per 1000 population representing a 21% decline between 2010 and 2015. Malaria parasite prevalence in children under five also registered a decline from 43% in 2010 to 33% in 2014 and 24% in 2017 [19]. The declining cases can be attributed to the Malawi government and donor efforts whereby, in 2010, the Malawi government introduced indoor residual spraying (IRS) in various districts. Furthermore, in 2012 and 2014, there was a distribution of free, long-lasting insecticide-treated nets (LLITN) to children and pregnant women in the country. The latter interventions have contributed to the reduction in the disease, however, the disease has not been contained [11].

The entire population of Malawi is at risk of malaria. However, pregnant women, their unborn babies and children under five years of age are at high risk of contacting the disease [19]. Children under five years bear negative consequences of the disease because when children are born, for about 6 months after birth, antibodies acquired from the mother during pregnancy protect the child; however, in the long run the child begins to lose the immunity as they grow by developing their own immunity to malaria. Therefore, they are prone to severe malaria infection because they lack acquired immunity [20]. Malaria continues to be a major public health problem in Malawi and a sustainable solution has not been found on how best to treat and eradicate the disease in different locations of Malawi. Therefore, in order to enhance strategies on how best to contain the disease, there is a need to look at the morbidity and mortality trends as well as forecasting future trends of the disease’s incidence.

## 2. Materials and Methods

### 2.1. Study Setting and Design

Nsanje is a district located in the southern part of Malawi with total land area of 1942 square kilometers. The district lies in the lower Shire River. The zone lies between 16 degrees southern latitude and 35 degrees eastern longitude. It has an estimated population of 299,168 of which 143,578 are male and 155,590 are female [16]. The study was a retrospective study where data from government Out Patient Department (OPD) registers for Nsanje district hospital were used to determine trends of malaria from 2015 to 2019 in Nsanje district. The target population for the study were children five years and below suspected to have malaria attending the OPD department at Nsanje District Hospital from January 2015 to December 2019. A sample of 420 children was collected.

### 2.2. Geographic Location of Nsanje District

**Figure 1:**
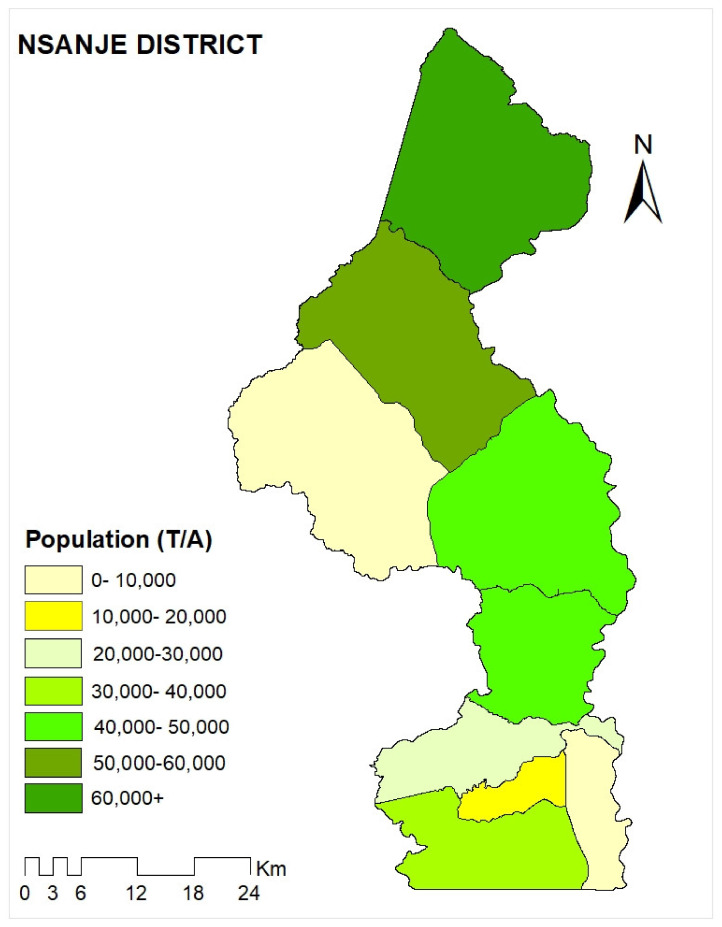
Map of Nsanje.

Figure 1 below shows the population density of Nsanje district.

### 2.3. Source of Data and Procedure

In order to determine the presence of plasmodium infection (malaria), blood smears from children were examined by a microscope in the laboratory by technicians. In the absence of laboratory technicians, rapid diagnostic tests were carried out by nurses to diagnose whether a child had malaria or not.

Five-year data (2015–2019) of children five years and below were extracted from the OPD government registers of Nsanje district hospital. Data that were extracted included the number of five years and under children with malaria diagnosed by month, deaths from malaria, treatment status whether new or revisiting, social demographics such as age and sex of the child. Data were extracted by an experienced coordinator for the hospital. Data related to the population at risk were collected from DHIS2 catchment population estimates.

### 2.4. Data Analysis and Procedure

The data that were extracted from the registers were entered in Microsoft Excel and analysis was conducted using Stata version 16.0. Descriptive statistics were carried out to calculate frequencies and percentages of overall malaria cases and trends of malaria transmission in terms of years, gender and age. Malaria morbidity was assessed using the incidence rate of malaria cases per 1000 population at risk and analyzed by years. Trends of malaria cases were summarized by years. Test positivity rate of the sample registered and the population at risk in Nsanje was used to calculate the incidence and death rate. The incidence rate was calculated by taking the total number of new cases in a given year and dividing it by the total sum of the population at risk and multiplying by 1000. The annual death rate was calculated by taking the total number of deaths in a particular year divided by the total population in the particular year and multiplying by 100,000. Findings were summarized using tables and line graphs. Furthermore, it should be noted that data related to the population at risk were collected from DHIS2 catchment population estimates.

Time series analysis was performed using a Seasonal Autoregressive Integrated Moving Average (SARIMA) model, which was applied to the malaria incidence data. SARIMA model can be expressed as SARIMA (p,d,q) (P,D,Q)_s_ where letters (p,d,q) are orders of autoregression, the order of difference and order of moving average, respectively; letters (P,Q,D)_s_ are the order of seasonal autoregression, the order of difference and the order of moving average, respectively, and s is the specific value of the cycle, which in this case is 12 [13]. Monthly time series plot of incidence (per 1000 population) was drawn to check for stationarity. The model was constructed according to autocorrelation function (ACF) and partial autocorrelation function (PACF) of model residuals. Then, ACF and PACF of estimating residuals were tested by Ljung–Box Q-test, and the model with the most significant coefficient, lowest AIC and lowest BIC was selected.

## 3. Results

### 3.1. Trends of Malaria Morbidity and Mortality from 2015 to 2019

A total of (420) cases of malaria in children under five were sampled from Nsanje district hospital within a period of 5 years (2015 to 2019). The majority of the cases were admitted to hospital (*n* = 317, 75.5%), while 103 (24.5%) were not admitted. The highest number of malaria cases was observed in 2016, and the least number of cases was observed in 2017. However, there was a constant number of malaria cases in 2015, 2018 and 2019 (Table 1).

**Figure 2:**
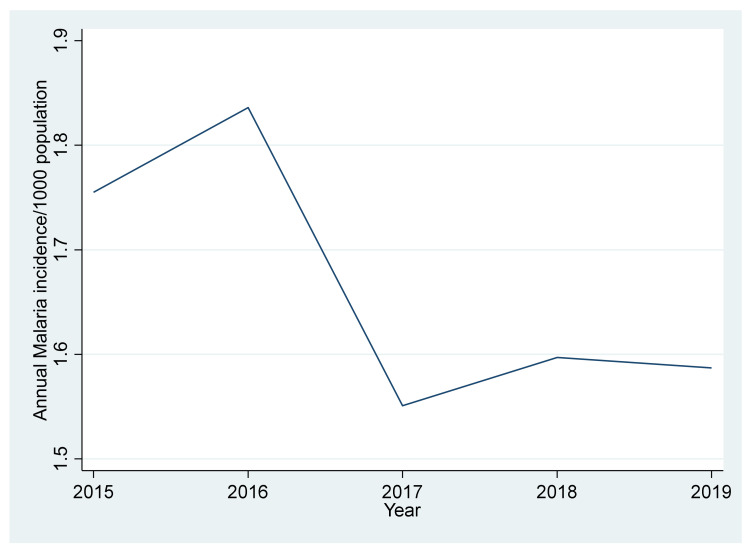
Trends of annual incidence of Malaria per 1000 population.

The overall mean annual incidence of malaria from 2015 to 2019 was 8.326 cases per 1000 population at risk. The incidence of malaria increased by 4.6% from 2015 to 2016, then it sharply decreased by 15.5% from 2016 to 2017. A slight increase by 2.9% from 2017 to 2018 was also observed and, lastly, it slightly decreased by 0.6% from 2018 to 2019 (Figure 2).

A total of 77 deaths were registered from the sample of 420 at the facility with mean annual death rate of 30.540 per 100,000 of the population at risk registered during the 5 year period. The overall malaria case fatality ratio was 0.92. The annual death rate decreased by 25.3% from 2015 to 2016, then the death rate increased by 35% from 2016 to 2017. From 2017 to 2018 the death rate decreased by 46.8% and a sharp increase by 88.7% was noticed from 2018 to 2019. The highest number of deaths from malaria, 19, was reported in 2019, seconded by 2017, which recorded 18 deaths. A slight decline in 2015 recording 17 deaths, while 2016 and 2018 recorded the lowest number of deaths 13 and 10, respectively. (Table 1).

Out of 420 malaria cases recorded from the sample in 5 years, 216 were from males representing 51.4% and 204 were females representing 48.6%. The annual incidence of malaria for males was 8.428 cases per 1000 population, while for females it was 7.259 cases per 1000 population as shown in Table 2.

Morbidity of malaria by age group was also analyzed: the majority of the cases (119) were reported among 24–35-month-old infants followed by those aged 36–47 months. Furthermore, those aged 0–11 months, 12–23 months and 48–69 months fell in between, respectively, and the least number of cases were among those aged 60 months.

**Figure 3:**
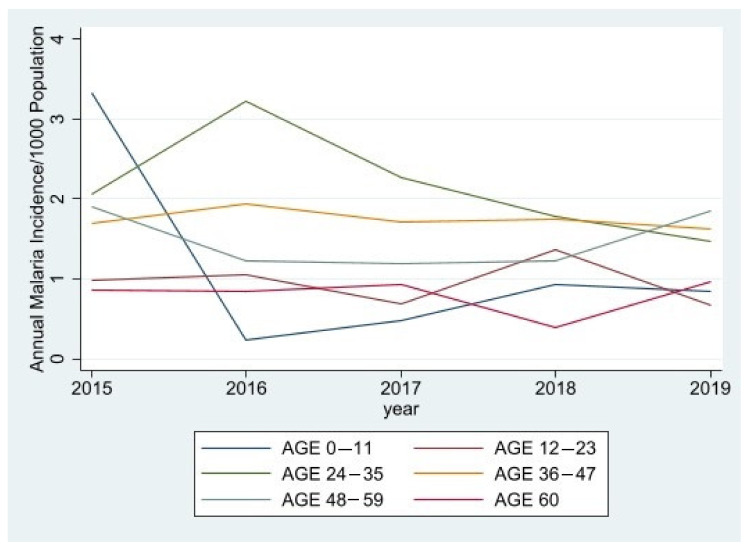
Annual incidence of Malaria for Age groups 0–5 years from 2015 to 2019.

Trends in annual incidence of malaria by age group are shown in Figure 3. The mean annual incidence of malaria in children aged five years and under also varied among different age groups. The incidence decreased by 18.5% between children aged 0–11 months and those aged 12–23 months. A sharp increase by 128% was noticed between those aged 12–23 months and 24–35 months. Sharp declines of 19.4% and 15% were observed between those aged 24–35 months and 36–47 months, and between those aged 36–47 months and 48–59 months, respectively. Those aged between 48–59 months and 60 months also had a decline of 46%.

### 3.2. Time Series Analysis

**Figure 4:**
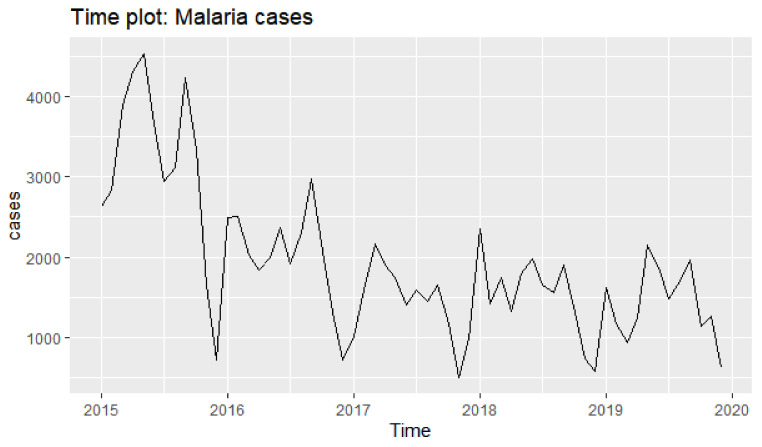
Time diagram for case incidence of malaria 2015–2019.

There were five years of observation from 2015 to 2029 used to construct the model and a time plot of the five years of observation is shown in Figure 4. The five years were used to evaluate the prediction effect. A forecast for each year using SARIMA (0, 1, 2) (0,1,1)_12_ for the next 3 years is shown in Supplementary Figure S1. From the results, the projected malaria cases were 15,676, 17,808 and 19,592 for the years 2020, 2021 and 2022, respectively. This suggests a fluctuating trend in malaria cases for the three years to come.

**Figure 5:**
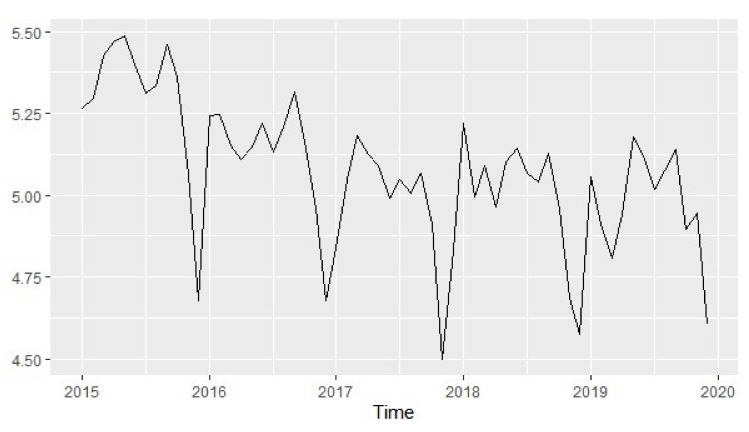
Box Cox transformed graph to stabilize the variance.

Box Cox transformation was performed to check if the graph has a stable variance and the result was −0.110, which was not close to one and, hence, the trend needed to be transformed in order to obtain a stable variance, Figure 5. The Augmented Dickey–Fuller Test indicated that the sequence was not stationery with *p* = 0.406, and, hence, there was a need for differencing both the trend and seasonality of the graph. 

**Figure 6:**
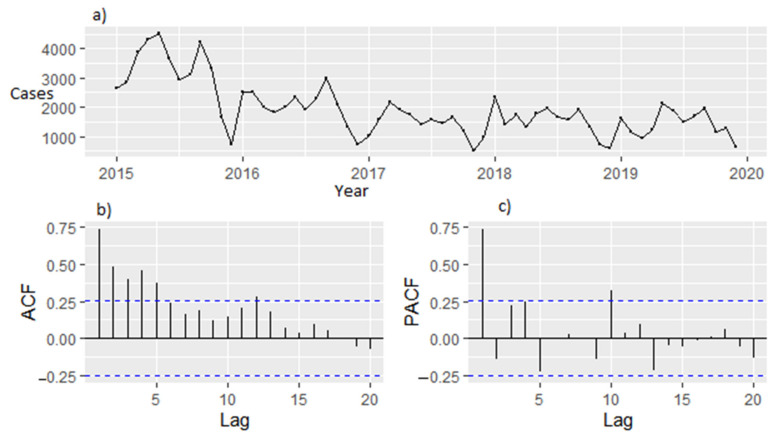
(**a**) Time diagram for ACF and PACF for estimating parameter before differencing: (**b**) ACF graph before differencing (d = 0, D = 0), (**c**) PACF graph before differencing (d = 0, D = 0).

**Figure 7:**
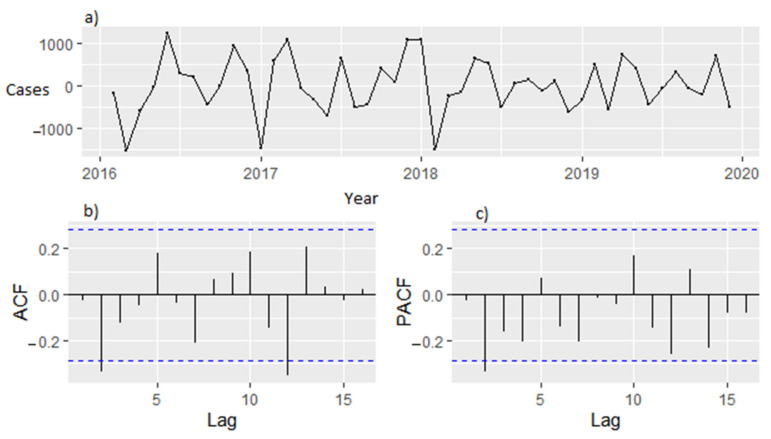
(**a**) Time diagram of malaria incidence after first trend and seasonal differencing. (**b**) ACF graph after trend and seasonal differencing (d = 1, D = 1) (**c**) PACF graph after trend and seasonal differencing (d = 1, D = 1).

Figure 6 shows the time diagram for ACF and PACF for estimating parameter before differencing. The trend (d = 0) and seasonal (D = 0). Figure 7 shows the time diagram for ACF and PACF for estimating parameter after differencing. The trend (d = 1) and seasonal (D = 1). 

### 3.3. Model Selection

The best model, SARIMA (0,1,2) (0,1,1) _12_, was selected to fit to the data. The conclusion to choose SARIMA (0,1,2) (0,1,1) _12_ was arrived at by looking at the model with the lowest AIC and BIC and the Ljung–Box Q-test of the model was valid (x^2 = 8.680, *p* = 0.467), indicating it was a white noise sequence. All parameter estimates were significant (Table 3). Therefore, the forecast is based on model SARIMA (0,1,2) (0,1,1)_12_.

## 4. Discussion

The purpose of the study was to analyze the epidemiology of malaria by looking at morbidity and mortality trends of malaria in children aged five and under over a period of 5 years. Malaria has been a major health problem in Malawi and it is considered as one of the disease burdens the country has been facing. Interventions of prevention, such as increased use of insecticides, the roll out of treatment and prevention efforts, have been successful in the country, as demonstrated by the increased use of insecticide-treated nets, improved access to prompt and effective treatment and the initiation of pilot studies of indoor residual spraying [19]. However, unlike other countries in the region, the recent data have not suggested a complete cure of the burden of disease [10].

The incidence rate depicted a fluctuating trend over the 5 years under study whereby 2016 had the highest incidence rate of 1.836 and the lowest incidence rate was in 2017 with 1.551 cases per 1000. These results are similar to the world malaria report 2019, which speculated on a decreasing trend of malaria case incidence in the sub-Saharan region with a decrease of 40% or more by 2020. In a study conducted by [21] in Kenya, it was found that 17% to 27% of all consultations among under five children were due to malaria, and 47% of admissions were due to malaria, with 63.4% of these admissions being children under five; the death rate was highest among children under five with 60.9 deaths per 1000 malaria under five admissions. The latter could be the case because of an increase in mosquito bites as the young children started sleeping with other children, leading to the improper utilization of mosquito bed nets. In addition, the Global Strategy for Malaria 2016–2030 highlights that the declining trend in malaria over the 5 years is due to the huge investment that has been put in place to completely eradicate the disease by 2030 [8]. Furthermore, nearly a quarter of the WHO funding in malaria goes to low income countries to completely eradicate the disease, and this further explains the decline in case incidence over the 5 years [22]. 

Annual death rate results showed that 2019 recorded the highest number of deaths (19) with an annual death rate of 35.888, and 2018 recorded the lowest number of deaths with an annual death rate of 19.012 per 100,000 population. Contrary to the results of this study, research conducted by [1] showed that most malaria deaths occurred in the high burden African regions and a declining trend was observed in countries such as Rwanda and Zambia. The latter results were attributed to the efficacy control strategies and favorable geographic positions of these countries. Since Nsanje district is a district located in the lower Shire and the climate all year through is favorable to the breeding of malaria, this might explain the reason why a fluctuating death rate trend is observed in the 5 year period.

The study also showed that there were more malaria cases in males (51.4%) than in females (48.6%). The annual incidence of malaria for males was 8.428 cases per 1000 population, while for females this was 7.259 cases per 1000 population. Morbidity of malaria by age group also showed that the majority of the cases (119) were reported among children aged 24–35 months, and the least number of cases were among those aged 60 months. These results are similar to a study conducted by [5], which highlighted that children in the age ranges of 2 to 5 months and 6 to 11 months are less likely to be diagnosed with malaria compared to children in the range from 24 to 59 months. This is as a result of the high immunity gained from breast milk in children who are still being breast fed. Furthermore, similar studies performed in Ethiopia and other parts of Africa [23] display the same results, where children who share the same bed with their mothers are more likely to be covered and sleep under a mosquito net than those who do not share a bed with their mothers. Another study in Rwanda by [24] supports the latter fact, where the study showed that malaria infection rates among children increase with increasing age because the use of mosquito nets reduces with the increasing age among children. This explains why, in the current study, there was a sharp increase in the malaria infection rate in children aged between (12–23 months) and (24–35 months) as these children are transitioning from sleeping with their parents to their siblings.

Malaria is linked to temperature and rainfall by many authors [25]. The time series analysis results show that malaria case incidence will have a fluctuating pattern for the two years projected. There will be a 4% decrease in case incidence from 2019 to 2020, a 3% increase from 2020 to 2021 and a 2% decrease from 2021 to 2022. These results show a cyclical pattern of malaria case incidence and the results are similar to those of [4]. There exist seasonal or annual cycles from mosquitoes/transmission patterns or the natural environment [26]. Therefore, the alternating incidence recorded yearly could be attributed to either people being careless about the disease or health care authorities not being consistent in the management of the disease [4].

## 5. Conclusions

The research was undertaken to depict trends of Malaria mortality and morbidity and to develop an appropriate model for forecasting future trends of malaria. It resulted that SARIMA(0,1,0)(1,1,0)_12_ was the appropriate model to fit the data.

The mortality and morbidity trend showed that malaria cases are growing at a fluctuating rate at Nsanje district hospital. Therefore, the Ministry of Health, together with the health providers, should intervene in the complete eradication of the disease by conducting more clinical trials on the disease. The model also proved that it was adequate to forecast monthly malaria cases at Nsanje district hospital. The model also had a good fit and, hence, was appropriate for the study. Therefore, Nsanje hospital should expect a fluctuation in malaria cases in the years to come.

## Figures and Tables

**Table 1:** Malaria Incidence and Death Rate from 2015 to 2019 Nsanje District.

Year	Population at Risk	No. of Cases (%)	No. of Deaths	Incidence Rate per 1000 Population	Death Rate per 100,000 Population	CFR in %
2015	47,864	84(20.0)	17	1.755	35.517	0.2
2016	49,059	90(21.4)	13	1.836	26.499	0.144
2017	50,303	78(18.6)	18	1.551	35.783	0.231
2018	52,597	84(20.0)	10	1.597	19.012	0.119
2019	52,943	84(20.0)	19	1.587	35.888	0.226

CFR—Case Fatality Rate.

**Table 2:** Distribution of Malaria Cases and Incidence Rate by Gender and Age from 2015 to 2019 in Nsanje District.

Variable	Category	Number (%)	Mean Annual Malaria Incidence per 1000 Population
Gender	Male	216(51.4)	8.428
	Female	204(48.6)	7.259
Age group	(0–11) months	40(9.5)	1.164
	(12–23) months	55(13.1)	0.948
	(24–35) months	119(28.3)	2.162
	(36–47) months	92(21.9)	1.742
	(48–69) months	75(17.9)	1.48
	(60) months	39(9.3)	0.799

**Table 3:** SARIMA model Selection.

Model	Estimate	t	p	Ljung–Box Q-Test		AIC	BIC	RMSE	MAPE
				Statistics	p				
SARIMA(0,1,0)(1,1,0)_12_	-	-	-	14.498	0.206	14.498	0.206	14.498	0.206
SAR1	−0.485	3.485	0.000	-	-	-	-	-	-
SARIMA(0,1,0)(0,1,1)_12_	-	-	-	17.857	0.0849	17.857	0.0849	17.857	0.0849
SMA1	−0.670	−2.490	0.012	-	-	-	-	-	-
SARIMA(0,1,1)(0,1,1)_12_	-	-	-	14.998	0.132	736.330	741.880	451.785	23.422
MA1	0.204	0.790	0.429	-	-	-	-	-	-
SMA1	−0.799	−1.600	0.110	-	-	-	-	-	-
SARIMA(0,1,2)(0,1,1)_12_	-	-	-	8.681	0.4670	726.420	733.820	403.909	21.447
MA1	−0.078	−0.610	0.542	-	-	-	-	-	-
MA2	−0.554	−4.652	3.292	-	-	-	-	-	-
SMA1	−0.730	−2.165	0.030	-	-	-	-	-	-

SARIMA: Seasonal Autoregressive Integrated Moving Average, AIC: Akaike Information Criteria, BIC: Bayesian Information Criteria, RMSE: Root Mean Squared Error, MAPE: Mean Absolute Percentage Error.

## Data Availability

Data for this study are available upon request from government registers in district hospitals.

## Supplementary Materials

The following are available online at https://www.mdpi.com/article/10.3390/ijerph182312784/s1, Figure S1: Forecast of Malaria case incidence from 2020 to 2022.
