# Supplementary figures and images for: Epidemiological Trends of Malaria in Five Years and under Children of Nsanje District in Malawi, 2015–2019
Source: Int J Environ Res Public Health. 2021 Dec 3;18(23):12784. doi: 10.3390/ijerph182312784 (PMC8657219; doi:10.3390/ijerph182312784)

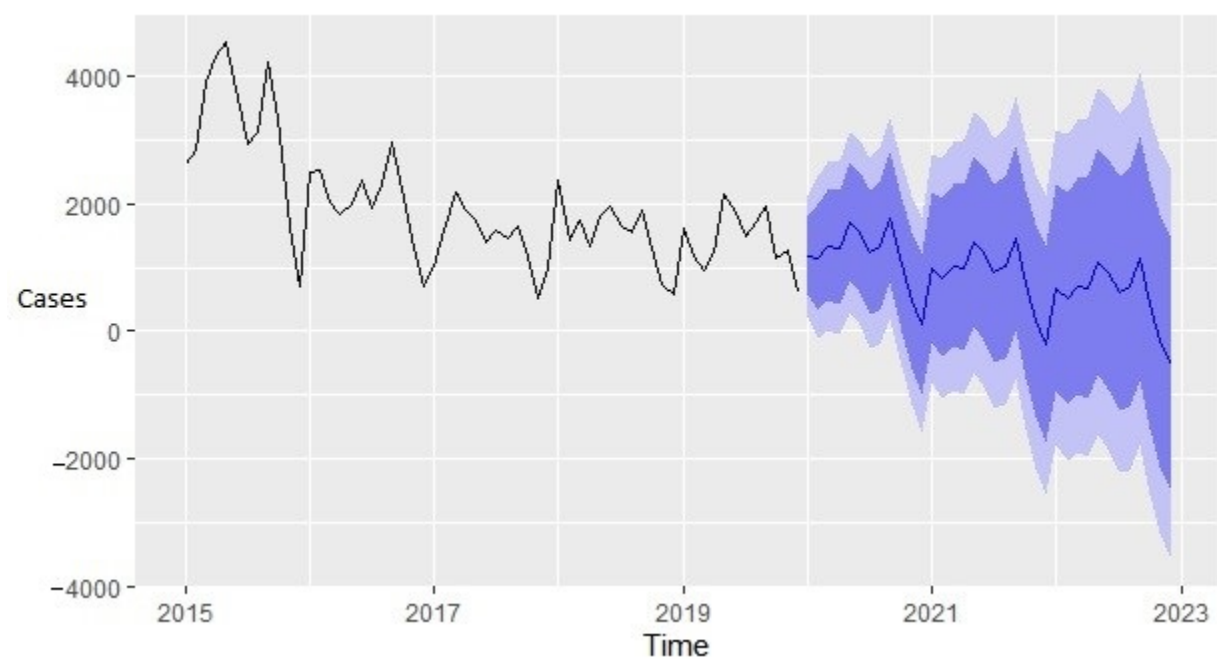

Supplement: Supplementary file 1 [file ijerph-18-12784-s001.zip › ijerph-1422580-supplementary.pdf]
